# Basophil Activation Test as Biomarker of Severity and Threshold of Allergic Reactions to Cow's Milk During Oral Food Challenges

**DOI:** 10.1111/all.70175

**Published:** 2025-12-18

**Authors:** Holly Boyd, Irene Bartha, Ru‐Xin Foong, Marta Krawiec, Andreina Marques‐Mejias, Hannah F. Marshall, Suzana Radulovic, Faye Harrison, Grammatiki Antoneria, Zainab Jama, Matthew Kwok, Ewa Pietraszewicz, Malak Eghleilib, Cristian Ricci, Tom Marrs, Gideon Lack, George Du Toit, Alexandra F. Santos

**Affiliations:** ^1^ Department of Women and Children's Health (Pediatric Allergy), School of Life Course Sciences, Faculty of Life Sciences and Medicine King's College London London UK; ^2^ Peter Gorer Department of Immunobiology, School of Immunology and Microbial Sciences King's College London London UK; ^3^ Children's Allergy Service, Evelina London Children's Hospital Guy's and St Thomas' Hospital London UK; ^4^ Africa Unit for Transdisciplinary Health Research (AUTHeR) North‐West University Potchefstroom South Africa

**Keywords:** anaphylaxis, basophil, basophil activation test, cow's milk allergy, food allergy, severity, skin prick test, specific IgE, threshold

## Abstract

**Background:**

Cow's milk allergy is the most common food allergy worldwide and the top cause of food anaphylaxis fatalities. Identifying patients at higher risk of severe symptoms as well as patients with a lower threshold of reactivity would improve their management. We aimed to assess the utility of putative biomarkers to identify these high‐risk patients.

**Methods:**

The severity of allergic reactions to baked milk (BM) and to fresh milk (FM) during oral food challenges (OFC) was assessed prospectively during the BAT2 study (NCT03309488), according to the Practall guidelines. Demographic, clinical and immunological parameters were compared between severe/non‐severe and higher/lower threshold reactors to BM or FM. Receiver Operating Characteristic curve analyses were performed to measure the accuracy of biomarkers with discriminative ability.

**Results:**

Seventy‐one children reacted to cow's milk: 22 (15%) to BM and 49 (43%) to FM. Seven (32%) and 12 (24%) reactors had severe symptoms during OFC to BM and FM, respectively. The median cumulative dose of milk protein tolerated was 0.44 g for BM and 0.143 g for FM. The basophil activation test (BAT) was the only biomarker that could distinguish severity and threshold groups. BAT optimal cut‐offs had 71% sensitivity and 100% specificity to identify severe reactors to BM and 96% sensitivity and 41% specificity to identify children reacting to 0.143 g or less of FM.

**Conclusions:**

BAT was the only biomarker for severity and threshold of allergic reactions to BM and FM, respectively. Once applied to clinical practice, BAT can help risk‐stratify cow's milk allergic patients and improve their management.

## Graphical abstract

The threshold of reactivity during fresh milk challenges was 70% lower than that of baked milk challenges, reflecting the higher allergenicity of fresh milk. BAT was the only biomarker statistically significantly different between severity and threshold groups, for baked milk and fresh milk allergies, respectively. Using optimal cut‐offs, sensitivity and specificity of BAT for severity of reactions to baked milk were 71/100% and for threshold of reactivity to fresh milk were 96%/41%. BAT, basophil activation test; IgE, immunoglobulin E; OFC, oral food challenge; SPT, skin prick test.

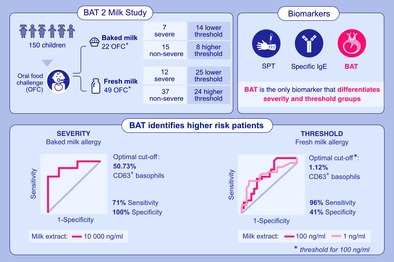

## 1 Introduction

Cow's milk allergy (CMA) is the most common food allergy worldwide, recently reported to affect about 6% of the general population in Western countries [1]. It resolves in about 60% of children by school age but can persist throughout life. Cow's milk is one of the most common causes of fatalities from food‐induced anaphylaxis, alongside peanut and other nuts [2, 3, 4]. It is extremely important to identify milk‐allergic patients at risk of developing potentially life‐threatening reactions or that might react to minute amounts of allergen, often through cross‐contamination. This risk stratification would allow clinicians to provide targeted education and preventive measures to ensure the safety of allergic patients and reduce the likelihood of allergic reactions during accidental exposure to their allergen. It may also serve to identify those food‐allergic patients eligible for disease‐modifying immunomodulatory treatments.

The basophil activation test (BAT) is a functional test that uses a patient's own live basophils and assesses whether they react to the allergen, using flow cytometry [5]. BAT has high diagnostic accuracy for peanut and other food allergies [6, 7, 8, 9]. It has also been demonstrated to reflect the severity and threshold of allergic reactions to peanut and egg [10, 11, 12, 13], with a higher proportion of basophils degranulating following allergen stimulation being associated with more severe symptoms and the allergen concentration at which basophils react in vitro being correlated to the dose at which patients react during their oral food challenge (OFC) to these foods. However, there is currently no reliable biomarker for severity or threshold of allergic reactions to cow's milk.

The BAT2 study (NCT03309488) was a diagnostic study, designed according to the STARD guidelines [14], which had the accuracy of the BAT to diagnose food allergy, including baked milk (BM) and fresh milk (FM) allergies, as the primary outcome. The BAT2 study included OFCs for all study participants. For cow's milk allergy, participants initially underwent a baked milk (BM) OFC and those who passed the BM OFC went on to have a FM OFC. The severity of allergic reactions and medication used to treat symptoms were recorded in real time as well as the doses of food administered and tolerated up until the allergic reaction. The utility of BAT and other tests to predict the severity and threshold of allergic reactions to BM and FM were secondary, but important, outcomes of the BAT2 study.

## 2 Methods

### 2.1 Study Design

The BAT2 study was registered at clinicaltrials.gov with NCT03309488. Eligibility criteria have been previously reported [15] and are listed here. Eligible participants were aged 6 months to 15 years, needed an OFC to cow's milk to clarify their allergic status to cow's milk. They either had: (1) a history of an immediate‐type allergic reaction to cow's milk or (2) no history of uneventful cow's milk consumption and/or (3) evidence of IgE sensitisation as documented by skin prick test (SPT) and/or serum specific IgE (sIgE). Exclusion criteria were: clinically significant chronic illness other than atopic diseases; a previous history of severe life‐threatening reaction to the suspected food with documented decrease in oxygen saturation (< 90%), hypotension (≥ 20% reduction in systolic blood pressure) and/or admission to intensive care; unwillingness to comply with study procedures, namely to undergo OFC; contraindication for OFC (such as uncontrolled atopic diseases, chronic medical conditions that pose significant risk in the event of anaphylaxis or treatment of anaphylaxis, inability to discontinue medications that might interfere with assessment or safety, treatment within 7–14 days with systemic steroids or prolonged high‐dose systemic steroids or immunosuppressants); current treatment with omalizumab, food allergen immunotherapy or other systemic immunomodulatory treatments; and inability to stop antihistamines prior to SPT.

All participants attended for BM OFC and the ones who did not react to BM were also offered a FM OFC. In total, 71 children reacted to cow's milk: 22 to BM and 49 to FM. The severity of allergic reactions was classified using 3 different approaches: the Practall criteria, the Ewan & Clark criteria and adrenaline use [16, 17] (Table S3). For analyses relating severity outcomes with other parameters, namely biomarkers, the Practall classification of severity was adopted. Medication used to treat allergic reactions, the cumulative dose of food protein tolerated and the dose eliciting a reaction were recorded for each patient. Patients underwent skin prick testing (SPT) and blood collection for specific immunoglobulin E (IgE) and BAT on the same day or within 6 months of their OFC.

The study was approved by the London–Westminster Research Ethics Committee (ref 17/LO/0296) and the UK Health Research Authority. Informed consent was obtained from a parent or guardian and assent obtained from the child before any study procedures.

### 2.2 Study Procedures

All children had SPT and blood collected for specific IgE and BAT, as previously described [15]. SPT was done using a single‐headed metal lancet; histamine dihydrochloride and 50% glycerol in buffered saline as positive and negative controls, respectively; and cow's milk extract (ALK Abello, Madrid, Spain), FM and BM slurry. The SPT result was calculated as the average of two perpendicular diameters including the longest one and recorded after 15 min. Serum total IgE, sIgE to cow's milk, boiled milk, alpha‐lactalbumin (Bos d 4), beta‐lactoglobulin (Bos d 5) and casein (Bos d 8) were determined using ImmunoCAP (Thermofisher, Uppsala, Sweden).

BAT was performed using whole blood incubated with milk extract (ALK‐Abello), baked milk using milk protein (Sigma‐Aldrich, Poole, UK) in solution heated at 180 degrees Celsius for 20 min, anti‐IgE (1 μg/mL, Sigma‐Aldrich, Poole, United Kingdom), formyl‐methionyl‐leucylphenylalanine (fMLP, 1 μM, Sigma‐Aldrich), diluted in RPMI (GIBCO, Paisley, United Kingdom), or RPMI alone. After incubation at 37°C for 30 min, samples were placed on ice and cold EDTA was added to stop degranulation. Samples were stained with CD123‐FITC, CD203c‐PE, HLA‐DR‐PerCP and CD63‐APC (all Biolegend, San Diego, CA). Red blood cells were lysed with Pharmlyse (BD Biosciences, San Diego, CA). Flow cytometry was performed using FACS Fortessa with FACSDiva software (BD Biosciences, San Jose, CA) and analysed using FlowJo software (version 7.6.1; TreeStar, Ashland, Ore). Basophils were gated as SSC^low^/CD203c+/CD123+/HLA‐DR‐ [11, 17]. Basophil activation was expressed as %CD63+ basophils and SI CD203c. Non‐responder basophils were defined as %CD63+ basophils below 5% to anti‐IgE and allergen.

Children aged 12 months or older had a double‐blinded placebo‐controlled food challenge (DBPCFC). Open incremental OFCs were done for children younger than 12 months of age to improve feasibility as open challenges require fewer doses and smaller volumes of food. Table S1 shows the dose regimens for OFC to BM and FM for different age groups. Of note, two additional low doses were incorporated in the dosing regimen for OFC considered to be high‐risk by the clinical team, with criteria including: history of severe allergic reaction or food‐protein induced enterocolitis syndrome in the last 12 months to cow's milk; history of anaphylaxis to the challenge food; clinical diagnosis of asthma or recurrent wheezing; hospitalisation or oral steroid rescue therapy required for asthma exacerbation in last 12 months; and SPT of 8 mm or greater to the challenge food. The outcome of the OFC and the severity of allergic reactions were determined according to the Practall guidelines [16], Ewan and Clark [18] and adrenaline use—Tables S2 and S3.

### 2.3 Statistical Analyses

Based on previous studies conducted by our group [7], a sample size of approximately 70 individuals is sufficient to investigate the performance of a diagnostic test under likely distributions of true versus false positive and negative tests. For example, under the hypothesis of relatively good diagnostic performances (< 10% of false positive and false negative) we may expect a sensitivity and specificity value of approximately 91% with a 15% confidence limit range (type‐I error = 5%).

For comparison of parameters across severity, patients with positive OFC were divided according to whether they had severe reactions during BM or FM OFC, based on the Practall classification. The threshold groups were formed based on the median cumulative dose eaten and tolerated (0.44 g for BM and 0.143 g for FM). Categorical variables were compared with Chi‐Square or Fisher's Exact Test, and continuous variables were compared with Mann–Whitney *U* Test between severity and threshold groups.

Receiver operator characteristic (ROC) curve analyses were performed to assess the accuracy of the various tests to identify severe and low threshold reactors to either BM or FM. Optimal cut‐offs were determined by the Youden index, compared with the dichotomy severe/non‐severe or lower/higher threshold. Positive and negative cut‐offs were defined by the highest point in the ROC curve with 100% sensitivity and the lowest point in the same curve with 100% specificity. All statistical evaluations were performed by SPSS version 27. Statistical tests were two‐tailed, and the type‐I error rate was set according to the Benjamini‐Hochberg correction based on a type‐I error of 5% (*α* = 0.05).

## 3 Results

### 3.1 Severity and Threshold of Allergic Reactions to Baked and Fresh Milk

In the BAT2 milk study, 71 children reacted to cow's milk: 22 to BM and 49 to FM. Among cow's milk allergic patients with a positive OFC, 7 (32%) and 12 (24%) had severe reactions during OFCs to BM and FM, respectively, according to the Practall classification. Figure S1 and Table S4 represent the distribution of symptom severity in BM and FM OFC, according to the Practall classification. Five (23%) and 6 (12%) patients reacting to BM and FM, respectively, received intra‐muscular adrenaline as part of the treatment of allergic reactions during OFC (Table 1). This included one patient who did not classify as a severe reactor to baked milk. No patient required more than one dose of adrenaline.

**TABLE 1:** Treatments administered during oral food challenges (OFC) performed as part of the BAT2 milk study.

Medication	Positive OFC to baked milk (*n* = 22)	Positive OFC to fresh milk (*n* = 49)
Adrenaline (intramuscular)	5 (23%)	6 (12%)
1 dose	5 (23%)	6 (12%)
2 doses	0 (0%)	0 (0%)
Anti‐histamines H1 or H2 (systemic)	22 (100%)	48 (98%)
Corticosteroids (systemic)	3 (14%)	1 (2%)
Salbutamol (inhaled)	5 (23%)	0 (0%)
Oxygen	4 (18%)	0 (0%)
Intravenous fluids	1 (5%)	0 (0%)

**FIGURE 1:**
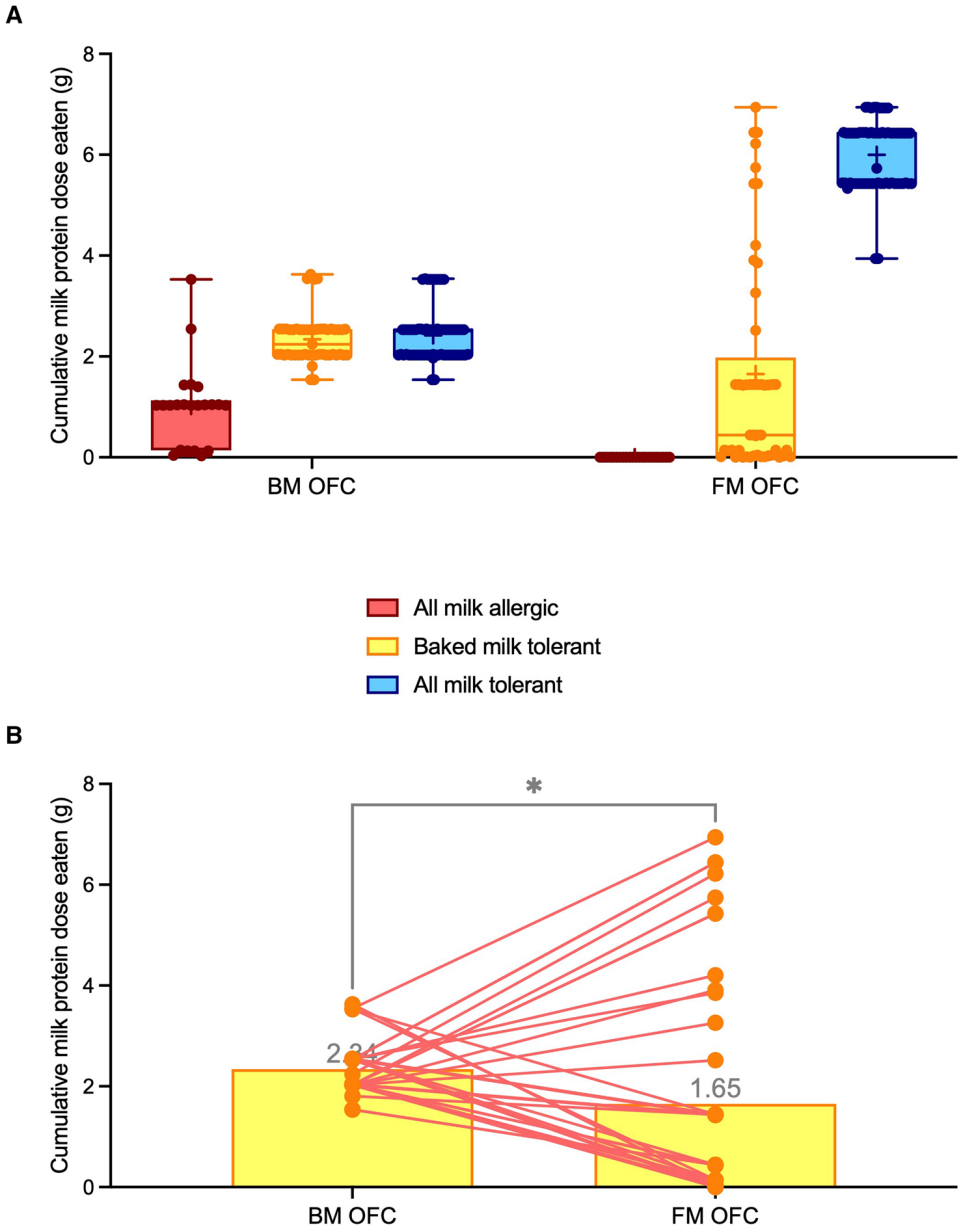
Milk allergic children tolerating baked milk react to fresh milk with a lower threshold than their own baked milk threshold. Threshold of reactivity expressed as cumulative milk protein eaten and tolerated in baked milk (BM) and fresh milk (FM) oral food challenges (OFC) in three phenotypes of milk allergy (A) and in patients who underwent two challenges, i.e., baked milk tolerant children (B). The median cumulative tolerated dose of baked milk was 2.34 g and of fresh milk was 1.65 g (*p = 0.026), Wilcoxon test.

In all reactors, the threshold dose of reactivity was 0.44 g of milk protein for BM and 0.143 g of milk protein for FM. Only 2 (9%) patients reacted to more than 2 g of cow's milk protein during BM OFC (Figure 1A). Patients who tolerated BM underwent OFC to FM. In patients who had both BM and FM OFC, the median cumulative dose of milk protein tolerated was lower for FM than for BM (1.65 versus 2.34, *p* = 0.026, Wilcoxon test), reflecting the known higher allergenicity of FM compared to BM (Figure 1B).

### 3.2 Biomarkers of Severity and Threshold

**FIGURE 2:**
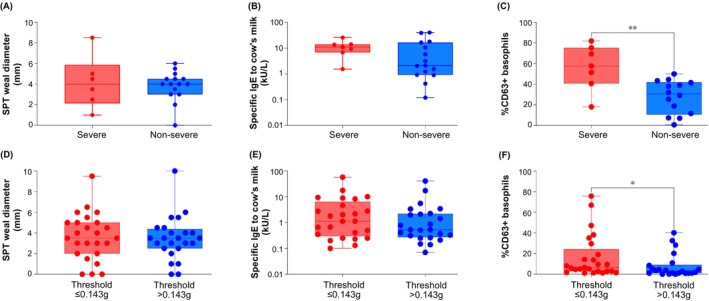
Skin prick test (SPT) to cow's milk extract, specific IgE to cow's milk and basophil activation test (BAT) results for patients with severe and non‐severe reactions during baked milk challenges (A, B, C) and for patients reacting to ≤ 0.143 g of milk protein on fresh milk challenges (D, E, F). Mann Whitney‐*U* test was used to compare the groups; *p < 0.05; **p < 0.01.

We compared demographic, clinical and immunological parameters between severe and non‐severe reactors to BM and FM, according to the Practall classification. No demographic or clinical element distinguished the two groups of BM reactors (Table 2, 2.1.; Table S5). Interestingly, patients considered high‐risk by the clinical team conducting the OFC were equally distributed between Practall severe and non‐severe reactors. Figure 2 shows the results of SPT, sIgE and BAT results for the BMA severity and FMA threshold groups. Only the BAT, with unstimulated basophils and following stimulation with milk extract at various concentrations from 10 to 10,000 ng/mL, emerged as statistically significantly higher in severe reactors compared to non‐severe reactors. Severe reactors to FM more frequently had a history of reacting to BM and had a higher threshold dose compared to non‐severe reactors (Table 2, 2.1.; Table S5).

**TABLE 2:** Demographic, clinical and immunological features in children with allergic reactions to baked or fresh cow's milk.

2.1. Severe versus non‐severe reactors during OFC to baked or fresh milk, as defined by the Practall classification			
(A) Baked milk			
Demographic, clinic and immunological characteristics	Severe reactors (*n* = 7)	Non‐severe reactors (*n* = 15)	*p*
Age (years)	4.1 (3.6; 8.8)	4.4 (1.5; 6.2)	0.581
Gender (females, %)	4 (57%)	3 (20%)	0.145
Ethnicity			
White	5 (71%)	7 (47%)	
Black	0 (0%)	3 (20%)	
Asian	2 (29%)	1 (7%)	0.241
Mixed	0 (0%)	3 (20%)	
Other	0 (0%)	1 (7%)	
History of allergic reaction to milk			
Baked milk	1 (14%)	6 (40%)	0.350
Fresh milk	7 (100%)	10 (67%)	0.135
Any milk	7 (100%)	12 (80%)	0.523
Atopic eczema	6 (86%)	13 (87%)	1.0
Other food allergies	7 (100%)	13 (87%)	0.598
Allergic rhinitis	4 (57%)	2 (13%)	0.054
Asthma	3 (43%)	2 (13%)	0.274
High‐risk doses given during DBPCFC (yes, %)	2 (29%)	6 (40%)	1.0
Time to reaction (min)	8 (0; 29)	10 (4; 23)	1.0
Cumulative dose tolerated (g)	0.43 (0.03; 0.44)	0.43 (0.43; 0.44)	0.407
Eliciting dose (g)	0.60 (0.03; 0.60)	0.60 (0.60; 0.60)	0.332
SPT to milk extract (mm)	4 (2; 6)	4 (3; 5)	0.970
SPT to fresh milk (mm)	7 (5; 9)	8 (5; 10)	0.910
SPT to challenge food slurry (mm)	3 (2; 5)	6 (3; 7)	0.078
Specific IgE to Boiled Milk (kU_A_/L)	5.96 (5.35; 16.5)	2.31 (0.93; 13.4)	0.267
Specific IgE to cow's milk (kU_A_/L)	11.0 (6.82; 14.1)	2.11 (0.92; 16.6)	0.210
Specific IgE to Bos d 4 (kU_A_/L)	4.0 (0.64; 5.49)	0.38 (0.04; 2.25)	0.056
Specific IgE to Bos d 5 (kU_A_/L)	1.22 (0.57; 6.23)	0.20 (0.01; 1.45)	0.080
Specific IgE to Bos d 8 (kU_A_/L)	8.44 (2.98; 16.93)	2.23 (0.80; 12.8)	0.132
BAT to milk extract at 100 ng/mL (%CD63+ Basophils)	37.0 (22.7; 59.9)	19.5 (12.0; 29.6)	**0.020**
BAT to milk extract at 10,000 ng/mL (%CD63+ Basophils)	57.63 (40.61; 75.38)	30.62 (13.61; 42.02)	**0.007**

(B) Fresh milk			
Demographic, clinic and immunological characteristics	Severe reactors (*n* = 12)	Non‐severe reactors (*n* = 37)	*p*
Age (years)	4.2 (2.2; 5.9)	4.2 (2.5; 7.9)	0.436
Gender (females, %)	8 (67%)	16 (43%)	0.196
Ethnicity			
White	8 (67%)	22 (60%)	
Black	1 (8%)	0 (0%)	
Asian	1 (8%)	4 (11%)	0.596
Chinese	1 (8%)	4 (11%)	
Mixed	1 (8%)	6 (16%)	
Other	0 (0%)	1 (3%)	
History of allergic reaction to milk			
Baked milk	8 (67%)	9 (24%)	**0.013**
Fresh milk	9 (75%)	30 (81%)	0.690
Any milk	12 (100%)	32 (87%)	0.315
Atopic eczema	10 (83%)	24 (65%)	0.298
Other food allergies	11 (92%)	35 (95%)	0.596
Allergic rhinitis	3 (25%)	13 (35%)	0.726
Asthma	2 (17%)	10 (27%)	0.703
High‐risk dose given during DBPCFC (yes, %)	9 (75%)	23 (62%)	0.503
Time to reaction from eliciting dose (min)	13 (6; 15)	12 (8; 24)	0.428
Cumulative dose tolerated (g)	0.44 (0.21; 1.44)	0.13 (0.00; 0.44)	**0.023**
Eliciting dose (g)	1 (0.48)	0.3 (0.01; 1.0)	**0.046**
SPT to milk extract (mm)	4 (3; 4)	4 (2; 5)	0.770
SPT to fresh milk (mm)	8 (6; 9)	6 (5; 8)	0.155
SPT to challenge food (mm)	5 (3; 6)	5 (3; 8)	0.683
Specific IgE to boiled milk (kU_A_/L)	0.39 (0.23; 0.70)	0.84 (0.19; 2.97)	0.429
Specific IgE to cow's milk (kU_A_/L)	0.52 (0.35; 2.03)	1.12 (0.25; 4.05)	0.625
Specific IgE to Bos d 4 (kU_A_/L)	0.21 (0.01; 1.23)	0.21 (0.02; 2.90)	0.771
Specific IgE to Bos d 5 (kU_A_/L)	0.51 (0.10; 1.50)	0.24 (0.08; 0.73)	0.389
Specific IgE to Bos d 8 (kU_A_/L)	0.18 (0.02; 0.29)	0.49 (0.14; 2.07)	0.060
BAT to milk extract at 100 ng/mL (%CD63+ Basophils)	4.45 (1.0; 20.18)	4.89 (1.38; 13.04)	0.951
BAT to milk extract at 10,000 ng/mL (SI CD203c)	1.28 (1.12; 1.58)	1.47 (1.10; 2.35)	0.079

2.2. Lower versus higher threshold of reactivity to baked or fresh milk during OFC			
(A) Baked milk			
Demographic, clinic and immunological characteristics	Threshold </=0.44 g (*n* = 14)	Threshold > 0.44 g (*n* = 8)	*p*
Age (years)	3.8 (1.6; 5.1)	6.1 (4.2; 10.9)	0.651
Female gender (% )	4 (29%)	3 (38%)	1.0
Ethnicity			
White	8 (57%)	4 (50%)	
Black	2 (14%)	1 (13%)	
Asian	2 (14%)	1 (13%)	0.766
Mixed	2 (14%)	1 (13%)	
Other	0 (0%)	1 (13%)	
History of allergic reaction to milk			
Baked milk	4 (29%)	3 (38%)	0.273
Fresh milk	10 (71%)	7 (88%)	
Any milk	11 (79%)	8 (100%)	
Atopic eczema	11 (79%)	8 (100%)	0.273
Other food allergies	12 (86%)	8 (100%)	0.533
Allergic rhinitis	2 (14%)	4 (50%)	0.137
Asthma	1 (7%)	4 (50%)	0.582
Severe reactors	5 (36%)	2 (25%)	1.0
Time to reaction (min)	9 (5; 24)	8 (3; 28)	0.815
SPT to milk extract (mm)	4 (3; 5)	4 (3; 6)	0.595
SPT to fresh milk (mm)	8 (5; 9)	8 (5; 10)	0.750
SPT to challenge food (mm)	3 (2; 6)	5 (5; 8)	0.110
Specific IgE to boiled milk (kU_A_/L)	5.66 (0.98; 14.18)	4.44 (1.63; 15.52)	0.920
Specific IgE to cow's milk (kU_A_/L)	8.76 (1.16; 14.73)	4.46 (1.67; 15.84)	0.764
Specific IgE to Bos d 4 (kU_A_/L)	1.50 (0.02; 3.57)	0.35 (0.13; 3.89)	0.916
Specific IgE to Bos d 5 (kU_A_/L)	0.70 (0.03; 2.74)	0.57 (0.08; 2.53)	0.916
Specific IgE to Bos d 8 (kU_A_/L)	4.47 (0.88; 13.15)	2.71 (1.44; 15.77)	0.972
BAT to milk extract at 100 ng/mL (%CD63+ Basophils)	20.93 (16.70; 36.28)	27.0 (14.12; 37.39)	0.804
BAT to egg white extract at 10,000 ng/mL (SI CD203c)	4.12 (2.73; 5.68)	4.71 (3.70; 6.89)	0.076

(B) Fresh milk			
Demographic, clinic and immunological characteristics	Threshold < =0.143 g (*n* = 25)	Threshold > 0.143 g (*n* = 24)	*p*
Age (years)	3.3 (2.1; 7.6)	5.8 (2.8; 8.2)	0.114
Gender (% females)	14 (56%)	10 (42%)	0.396
Ethnicity			
White	17 (68%)	13 (54%)	
Black	1 (4%)	0 (0%)	
Asian	2 (8%)	3 (13%)	0.691
Chinese	2 (8%)	3 (13%)	
Mixed	3 (12%)	4 (17%)	
Other	0 (0%)	1 (4%)	
History of allergic reaction to milk			
Baked milk	8 (32%)	9 (38%)	0.769
Fresh milk	23 (92%)	16 (67%)	**0.037**
Any milk	24 (96%)	20 (83%)	0.189
Atopic eczema	13 (52%)	21 (88%)	**0.012**
Other food allergies	24 (96%)	22 (92%)	0.587
Allergic rhinitis	6 (24%)	10 (42%)	0.232
Asthma	5 (20%)	7 (29%)	0.520
Severe reactors	3 (12%)	9 (38%)	0.051
Time to reaction (min)	13 (8.5; 23)	11.5 (5.50; 21.5)	0.245
SPT to milk extract (mm)	4 (2; 5)	4 (3; 4)	0.630
SPT to fresh milk (mm)	7 (4; 8)	7 (5; 9)	0.595
SPT to challenge food slurry (mm)	5 (1; 8)	5 (4; 7)	0.470
Specific IgE to Boiled Milk (kU_A_/L)	0.62 (0.27; 2.57)	0.39 (0.16; 2.25)	0.509
Specific IgE to Cow's milk (kU_A_/L)	1.14 (0.30; 6.46)	0.53 (0.27; 2.22)	0.352
Specific IgE to Bos d 4 (kU_A_/L)	0.53 (0.04; 2.61)	0.15 (0.01; 2.57)	0.336
Specific IgE to Bos d 5 (kU_A_/L)	0.55 (0.12; 1.59)	0.19 (0.09; 0.44)	0.077
Specific IgE to Bos d 8 (kU_A_/L)	0.30 (0.15; 1.43)	0.27 (0.03; 2.01)	0.726
BAT to milk extract at 100 ng/mL (%CD63+ Basophils)	6.53 (2.83; 24.28)	3.23 (0.91; 9.09)	**0.017**
BAT to milk extract at 1 ng/mL (SI CD203c)	1.49 (1.13; 1.93)	1.14 (1.03; 1.28)	**0.013**

*Note:* Patients considered high‐risk were given 2 additional low doses (Table E1). *p* values refer to Mann–Whitney *U* test. Significant *p* values are highlighted in bold.

Abbreviations: anti‐IgE, anti‐immunoglobulin_E; BAT, basophil activation test; BM, baked milk; Ig, immunoglobulin; ME, milk extract; SPT, skin prick test.

In participants who underwent the FM challenge, participants with a lower threshold of reactivity to FM more frequently had a history of reacting to FM and had a lower prevalence of atopic eczema, compared with children with a higher threshold of reactivity. Interestingly, among children who reacted to FM, those with a lower threshold had a higher proportion of activated basophils following stimulation with milk allergen extract compared to higher threshold children (Table 2, 2.2.).

### 3.3 Basophil Activation Test as Biomarker of Severity for Baked Milk Allergy and of Threshold of Allergic Reactions to Fresh Milk

The BAT was the only biomarker that was statistically significantly different between severity and threshold groups, as defined by the Practall classification and the cumulative tolerated dose, respectively. However, this only applied to the severity of allergic reactions to BM and the threshold of reactivity to FM. We performed ROC curve analyses to define cut‐offs and measure their performance in identifying severe reactors and lower threshold reactors to BM for severity and FM for threshold.

**FIGURE 3:**
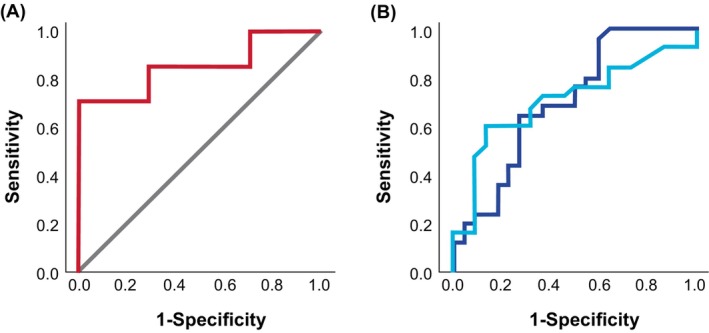
Receiver Operating Characteristic curve for the basophil activation test to predict severe reactions (using the Practall classification [16]) and their threshold (as determined by the cumulative dose eaten and tolerated) during oral food challenges. (A) Baked milk—BAT using %CD63+ Basophils at 10,000 ng/mL of milk extract to identify severity. (B) Fresh milk—BAT using %CD63+ Basophils at 100 ng/mL (darker blue) or SI CD203c at 1 ng/mL (lighter blue) of milk extract to identify threshold.

For severity of allergic reactions to baked milk, the proportion of CD63‐positive basophils following stimulation with 100 ng/mL or 10,000 ng/mL showed an area under the ROC curve of 0.816 (95% confidence interval (CI): 0.603; 1.030) and 0.857 (95% CI: 0.657; 1.058), respectively (Figure 3A; Figure S2). The optimal and 100% specificity cut‐off for BAT coincided and had 71% sensitivity with 100% positive predictive value (PPV) and 88% negative predictive value (NPV) with an overall accuracy in identifying severe reactors to BM of 90%. No patient with BAT below 14.85% CD63+ basophils at 10,000 ng/mL of milk extract developed severe reactions (Table 3; Table S6).

**TABLE 3:** Diagnostic performance of optimal, 100% sensitivity and 100% specificity cut‐offs for the best biomarkers for severity (as defined by the Practall classification) and threshold of allergic reactions to milk during OFC.

Severity										
Food and parameter	Cut‐off		AUC ROC	Sensitivity	Specificity	PPV	NPV	Accuracy	TP/FP	TN/FN
Baked milk %CD63+ Basophils 10,000 ng/mL ME	100% S	14.85	0.643	100%	71%	41%	100%	52%	7/10	4/0
	OPTIMAL	50.73	0.857	71%	100%	100%	88%	90%	5/0	14/2
	100% Sp									

Threshold										
Food and parameter	Cut‐off		AUC ROC	Sensitivity	Specificity	PPV	NPV	Diagnostic accuracy	TP/FP	TN/FN
Fresh milk %CD63+ Basophils 100 ng/mL ME	100% S	1.04	0.682	100%	36%	64%	100%	70%	25/14	8/0
	OPTIMAL Youden	1.12	0.685	96%	41%	65%	90%	70%	24/13	9/1
	100% Sp	43.71	0.560	12%	100%	100%	50%	53%	3/0	22/22

Abbreviations: AUC, area under the curve; BAT, basophil activation test; FN, false negative; FP, false positive; IgE, immunoglobulin E; NPV, negative predictive value; PPV, positive predictive value; ROC, receiver operating characteristic curve; S, sensitivity; Sp, specificity; TN, true negative; TP, true positive.

For threshold of allergic reactions to fresh milk, BAT showed an area under the ROC curve of 0.703 (95% CI: 0.551; 0.855) for both proportion of CD63‐positive basophils after stimulation with 100 ng/mL of milk extract and basophil stimulation index of CD203c after stimulation with 1 ng/mL of milk extract (Figure 3B). BAT's optimal cut‐off showed a 96% sensitivity and 41% specificity, 65% PPV, 90% NPV and overall accuracy of 70%. All patients with BAT above 43.71% CD63‐positive basophils at 100 ng/mL of milk extract reacted to 0.143 g or less of FM protein (Table 3; Table S6).

## 4 Discussion

Cow's milk allergy is the most common food allergy in childhood and the most common cause of food‐induced anaphylaxis, alongside peanut and tree nuts. Hence, identifying cow's milk allergic patients at risk of severe reactions or of accidental reactions to small amounts of milk (such as in cross‐contamination) is important to ensure optimal management. In the BAT2 milk study, where all children had milk OFC, we identified the BAT as the only biomarker able to distinguish severe from non‐severe reactors to BM, and lower from higher threshold reactors to FM. Once available in routine clinical practice, BAT would be informative to support risk stratification for improved management of cow's milk allergic patients.

This is the largest cow's milk allergy study, in which all participants underwent OFC and were tested with various biomarkers, including SPT, specific IgE and BAT to a variety of milk allergen preparations. The severity and threshold dose of allergic reactions were assessed and recorded prospectively in real‐time by the team performing the OFC. There was no complete agreement between the three severity classifications and between the severity according to Practall and administration of adrenaline. Specifically, there were 4 out of 7 and 6 out of 14 reactions to baked and fresh milk, respectively, which were treated with adrenaline, and 1 reaction which did not reach severity grade but required adrenaline as part of the treatment. The patients who were classified as severe but did not require adrenaline were mostly patients with intense symptoms but limited to skin, gastrointestinal tract or the upper airway. Conversely, the patient treated with adrenaline developed urticaria, persistent cough, tight chest and changed behaviour becoming quiet; whilst this may not tick the boxes for severity in the Practall classification, as a whole, it prompted the clinical team to treat with adrenaline. These discrepancies highlight the subjectivity of severity classification and the clinical judgement involved in the decision to treat with adrenaline, which may not be reflected in a paper chart.

None of the demographic and clinical parameters were different between severe and non‐severe reactors. Although we cannot exclude that the specific nature of the sample under analysis and its relatively small sample size, along with the large number of statistical tests, could have generated many false negative and false positive results, the use of correction for multiple comparisons adds robustness to our findings.

The severity and threshold of allergic reactions during OFC may arguably be an accurate measure of severity and threshold of allergic reactions that happen in the community as allergic reactions during OFC happen in ideal conditions (i.e., with atopic conditions in good control, no infections, minimised presence of other co‐factors, graded dosing schedule at set intervals and exposure halted at the first signs of an allergic reaction). This is often not the case in allergic reactions that occur in the community which may happen during eczema or asthma exacerbation or during an infection, with relatively higher doses of allergen exposure and possible presence of co‐factors, which can decrease the allergen threshold and increase the severity of allergic symptoms [19, 20]. However, the alternative to using allergic reactions that happen during OFC is to use reported reactions, with the potential inherent limitations, including recall bias.

The rate of severe reactions in our study was relatively lower than in other studies. There were fewer severe reactions during the FM OFCs compared to the BM OFCs, which could have been influenced by the fact that all children who were challenged to FM were tolerant and had already passed an OFC to BM. In addition, there was a higher number of patients who passed their baked milk/egg challenge and did not attend their FM/loosely cooked egg OFC (13 for milk versus 7 for egg). Some of these patients declined the additional OFC due to large SPT and fear of an allergic reaction. These factors, together with the time gap between blood collection and FM OFC, limit the granularity of biomarkers' data.

It was impressive that 85% of children challenged to BM tolerated it. It is known that most milk allergic patients tolerate BM; however, the BAT2 cohort is representative of the equivocal cohort that needs an OFC to determine whether they are allergic to milk and thus the proportion of BM tolerant children found was higher than previously anticipated. Interestingly, all but 2 BM allergic patients reacted to more than 2 g of milk protein (corresponding to 1.3 of a muffin), which indicates that 1 muffin is probably suitable for most BM OFCs to identify whether it is safe to eat BM at home. Importantly, BAT could be helpful in identifying such patients without the need for OFC, as we previously demonstrated [21]. For the children who attended 2 OFCs, the cumulative dose tolerated during FM OFCs was lower than for BM OFCs. Despite the time gap between OFCs and the fact that children attending FM OFCs were tolerating BM, the threshold dose during FM OFCs was overall lower than that during BM OFCs. This reflects the higher allergenicity of FM compared to BM, which is probably due to less processing and the absence of the matrix effect of wheat in the food used for BM OFCs.

Previous studies have suggested specific IgE to casein as a possible biomarker of BM allergy [22, 23]. In our study, specific IgE to casein (Bos d 8) was higher in patients with severe reactions and low thresholds of reactivity to baked milk but this did not reach statistical significance. The levels of casein‐specific IgE were similar between severity and threshold groups to fresh milk. This suggests a potentially greater relevance of casein in the context of BM, rather than FM, allergy; however, the fact that only BAT reached statistically significant differences highlights the greatest value of functional, rather than titre‐based, tests in assessing risk of allergic reactions.

Interestingly, and possibly paradoxically, children with a lower threshold of reactivity had less severe reactions. The relationship between severity and threshold is not fully understood and can be controversial. There is a general belief that if someone is suddenly exposed to a larger amount of the allergen, they are at higher risk of severe allergic reactions. However, our findings suggest that patients who react after having consumed lower amounts of the allergen do not have as severe reactions as the ones who only react to higher doses, by which point the amount ingested is higher. We have also observed a similar association in a previously published egg allergy study [12]. Possibly, developing symptoms earlier in the dose escalation works as a warning sign and leads to stopping ingestion earlier and thus prevention of more significant reactions. Allergic reactions are a defence mechanism, after all. Nevertheless, these findings need to be reproduced in independent cohorts before they can be generalised.

We, and others, have previously shown higher basophil reactivity to milk allergens in patients allergic to BM compared to those who were BM tolerant [23]. In another study, by Rubio et al., assessing the utility of BAT to decide when to reintroduce milk in the diet of previously milk allergic children, BAT results were directly correlated with the severity of allergic reactions and inversely correlated with the eliciting dose of cow's milk during OFC [24]. Our study confirms the relevance of BAT in the context of risk stratification and goes beyond previous studies, in that our study included more patients, with DBPCFC in all children and determined cut‐offs and performance of BAT in identifying cow's milk allergic patients at higher risk. These findings are robust and can have high practical value, in the future, when BAT becomes available as a clinical test. However, this was a single centre study conducted in a tertiary care centre in the UK and, whilst it is likely to be representative of the cow's milk allergic children in other geographical locations, especially considering the diversity of our patient population, this needs to be confirmed.

Our study was the first to use BM preparation alongside a standard milk allergen extract. We prepared the BM by heating milk protein in solution in an oven at 180 degrees Celsius for 20 min. The possible effect of the wheat matrix was not tested as it is difficult to reproduce this in vitro, especially in the context of a test that aims to be used in routine clinical practice. The BAT2 study results showed that, when testing patients with suspected allergy to cow's milk, BAT can, not only confirm the diagnosis of BM and FM allergies [21], but also identify patients that are at a risk of reaction to small amounts of allergen and severe reactions. Although the comparator was OFC, which is different from accidental exposure in a real‐life setting, and the discriminative ability of BAT was modest, particularly for threshold and FM reactions, the information provided by BAT can add to the context of the individual patient. Patients with a high degree of basophil activation can be flagged for more intense education and follow‐up and, possibly, immunomodulatory therapies, such as cow's milk oral immunotherapy.

In summary, we present the results of a state‐of‐the‐art study of cow's milk allergy, in which all children had a BM OFC and most also had a FM OFC. The outcomes of OFCs were compared with demographic, clinical and immunological parameters. The BAT stood out as the only biomarker able to distinguish severe from non‐severe reactors and low from high threshold reactors, severity for BM allergy and threshold for FM allergy. BAT's performance was good, both in terms of sensitivity and specificity, better at predicting the severity of allergic reactions to BM than at predicting the threshold dose of reaction to FM, and could be used to identify high‐risk patients in clinical practice to support patients' management.

## Author Contributions

I.B., H.B., R.‐X.F., A.M.‐M., H.F.M., S.R., G.D.T. and A.F.S. performed study procedures related to patient recruitment and cared for study participants. M.K., Z.J., G.A. performed and analyzed the basophil activation test. F.H. and A.F.S. designed the food frequency questionnaires and oral food challenge protocols. F.H. and M.E. prepared the oral food challenge doses. M.E. and E.P. managed the study data and database. C.R. performed the statistical analyses. A.F.S. designed the study protocol and acted as chief investigator for the study, obtained and managed the research funding, supervised data acquisition, data management and data analyses, and wrote the first version of the manuscript. All authors critically reviewed the manuscript and approved its final version.

## Funding

This study was funded by the Medical Research Council through MRC Clinician Scientist Fellowship MR/M008517/1 and MRC Transition Fellowship MR/T032081/1 awarded to A.F.S.

## Conflicts of Interest

Dr Radulovic and Dr Marshall report salary support from grants from the National Institute of Allergy and Infectious Diseases (NIAID, NIH). Dr Lack reports grants from the National Institute of Allergy and Infectious Diseases (NIAID, NIH), others from Food Allergy & Research Education (FARE), others from MRC & Asthma UK Centre, others from UK Dept of Health through NIHR, others from National Peanut Board (NPB), others from The Davis Foundation, during the conduct of the study; he is a shareholder in DBV Technologies, and Mighty Mission Me, with personal fees from Novartis, Sanofi‐Genyzme, Regeneron, ALK‐Abello, personal fees from Lurie Children's Hospital, outside the submitted work. Dr Du Toit reports grants from the National Institute of Allergy and Infectious Diseases (NIAID, NIH), Food Allergy & Research Education (FARE), MRC & Asthma UK Centre, UK Department of Health through NIHR, Action Medical Research and National Peanut Board. Scientific Advisory Board member Aimmune. Investigator on pharma‐sponsored allergy studies (Aimmune and DBV Technologies). Scientific advisor to Aimmune, DBV and Novartis. Dr. Santos reports grants from Medical Research Council (MR/M008517/1; MC/PC/18052; MR/T032081/1), Food Allergy Research and Education (FARE), the Immune Tolerance Network/National Institute of Allergy and Infectious Diseases (NIAID, NIH), Asthma UK (AUK‐BC‐2015‐01), BBSRC, Rosetrees Trust and the NIHR through the Biomedical Research Centre (BRC) award to Guy's and St Thomas' NHS Foundation Trust, during the conduct of the study; personal fees from Thermo Scientific, Nutricia, Infomed, Novartis, Allergy Therapeutics, Buhlmann, Nestle, IgGenix, as well as research support from Buhlmann and Thermo Fisher Scientific through a collaboration agreement with King's College London. The other authors have nothing to disclose.

## Supporting information


**Figure S1.** Severity of symptoms experienced during challenges to baked milk (A, *n* = 22) and fresh milk (B, *n* = 49), assessed and classified in real‐time by the clinical team attending the oral food challenge. Scores from 0 to 3 are given depending on the severity of symptoms, according to the Practall guidelines 16.

Figure S1B.

**Figure S2.** Receiver Operating Characteristic curve for the basophil activation test to predict severe reactions during oral food challenges to baked milk using %CD63+ Basophils at 100 ng/mL of milk extract.

Appendix S1.

## Data Availability

The data that support the findings of this study are available on request from the corresponding author. The data are not publicly available due to privacy or ethical restrictions.
