# Supplementary figures and images for: Basophil Activation Test as Biomarker of Severity and Threshold of Allergic Reactions to Cow's Milk During Oral Food Challenges
Source: Allergy. 2025 Dec 18;81(4):1193–204. doi: 10.1111/all.70175 (PMC13040645; doi:10.1111/all.70175)

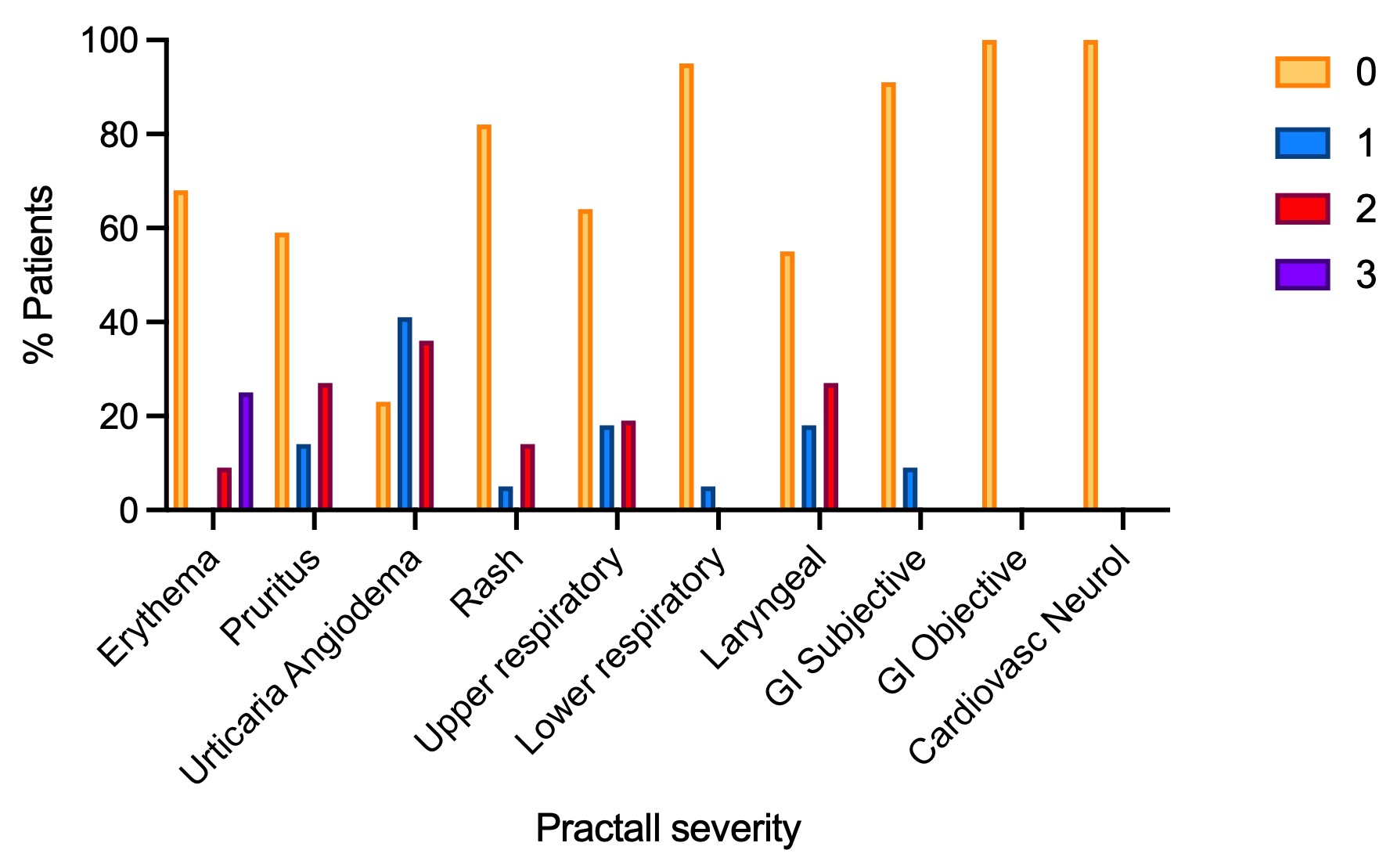

Supplement: Supplementary file 1 — Figure S1. Severity of symptoms experienced during challenges to baked milk (A, n = 22) and fresh milk (B, n = 49), assessed and classified in real‐time by the clinical team attending the oral food challenge. Scores from 0 to 3 are given depending on the severity of symptoms, according to the Practall guidelines 16. [file ALL-81-1193-s002.jpg]

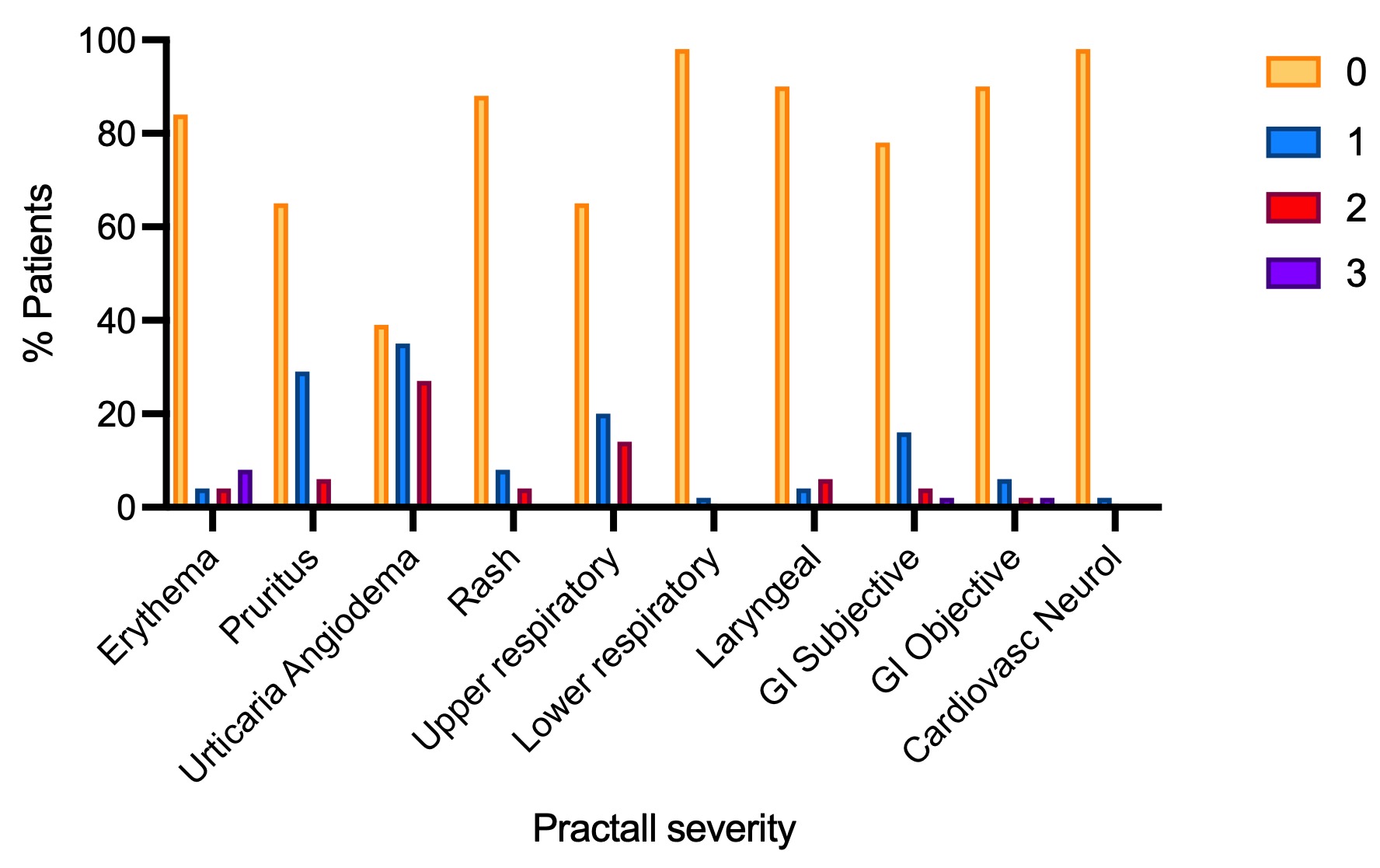

Supplement: Supplementary file 2 — Figure S1B. [file ALL-81-1193-s003.jpg]

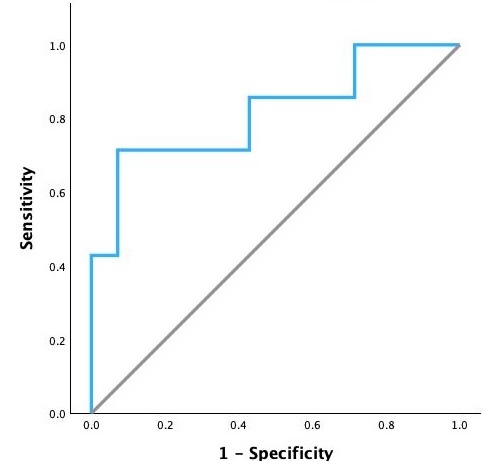

Supplement: Supplementary file 3 — Figure S2. Receiver Operating Characteristic curve for the basophil activation test to predict severe reactions during oral food challenges to baked milk using %CD63+ Basophils at 100 ng/mL of milk extract. [file ALL-81-1193-s001.jpg]
