# Supplementary material for: Basophil Activation Test as Biomarker of Severity and Threshold of Allergic Reactions to Cow's Milk During Oral Food Challenges
Source: Allergy. 2025 Dec 18;81(4):1193–204. doi: 10.1111/all.70175 (PMC13040645; doi:10.1111/all.70175)
Supplement: Supplementary file 4 — Appendix S1. [file ALL-81-1193-s004.docx]

**Supplementary material**

**Basophil activation test as biomarker of severity and threshold of allergic reactions to cow’s milk during oral food challenges**

**Short title**: Severity and threshold biomarkers of cow’s milk allergy

Ru-Xin Foong*, Holly Boyd*, Irene Bartha*, Marta Krawiec*, Andreina Marques-Mejias*, Hannah F. Marshall*, Suzana Radulovic*, Faye Harrison, Grammatiki Antoneria, Zainab Jama, Matthew Kwok, Ewa Pietraszewicz, Malak Eghleilib, Cristian Ricci, Tom Marrs, Gideon Lack, George Du Toit, Alexandra F. Santos

**shared first-authors*

^1^Department of Women and Children’s Health (Pediatric Allergy), School of Life Course Sciences, Faculty of Life Sciences and Medicine, King’s College London, London, United Kingdom

^2^Peter Gorer Department of Immunobiology, School of Immunology and Microbial Sciences, King’s College London, London, United Kingdom

^3^Children’s Allergy Service, Evelina London Children’s Hospital, Guy’s and St Thomas’ Hospital, London, United Kingdom

^4^Africa Unit for Transdisciplinary Health Research (AUTHeR), North-West University, Potchefstroom, South Africa

***Corresponding Author:**

Alexandra F. Santos

Address: Department of Paediatric Allergy, 2^nd^ floor, South Wing, St Thomas’ Hospital, SE1 7EH London, United Kingdom

Telephone number: +44 (0) 20 7188 0610

Email address: alexandra.santos@kcl.ac.uk

**Table E1.** Dose regimens in grams of protein for oral food challenges.

DBPCFC, double-blind placebo-controlled food challenge.

1. **Baked milk challenges.** *The initial doses will be given only in patients considered to be high-risk (HR). **Cumulative dose does not include the High-Risk doses

| Age | | 6-12 months | 1-3 years | 4-10 years | 11-16 years |
| --- | --- | --- | --- | --- | --- |
| Food | | BAKED COW’S MILK | | | |
| Type of challenge | | Open | DBPCFC | DBPCFC | DBPCFC |
| Number of placebo doses randomly interspersed | | 0 | 1 | 1 | 1 |
| Active doses  (g) | High Risk Dose 1* | 0.003 | 0.003 | 0.003 | 0.003 |
|  | High Risk Dose 2* | 0.01 | 0.01 | 0.01 | 0.01 |
|  | Dose 1 | 0.03 | 0.03 | 0.03 | 0.03 |
|  | Dose 2 | 0.1 | 0.1 | 0.1 | 0.1 |
|  | Dose 3 | 0.2 | 0.3 | 0.3 | 0.3 |
|  | Dose 4 | 0.4 | 0.6 | 0.6 | 0.6 |
|  | Open dose | 0.8 | 1.0 | 1.5 | 2.5 |
|  | **Cumulative protein dose**** | **1.53** | **2.03** | **2.53** | **3.53** |
|  | Total number of muffin/cookies | 1.0 | 1.3 | 1.6 | 2.2 |

**B. Fresh milk challenges.** Dose regimens in grams of protein for DBPCFC to fresh milk according to the different age groups.

*The initial doses will be given only in patients considered to be high-risk (HR). **Cumulative dose does not include the High-Risk doses.

| Age | | 6-12 months | 1-3 years | 4-10 years | 11-16 years |
| --- | --- | --- | --- | --- | --- |
| Food | | FRESH COW’S MILK | | | |
| Type of challenge | | Open | DBPCFC | DBPCFC | DBPCFC |
| Number of placebo doses randomly interspersed | | 0 | 1 | 1 | 1 |
| Active doses  (g) | High Risk Dose 1* | 0.003 | 0.003 | 0.003 | 0.003 |
|  | High Risk Dose 2* | 0.01 | 0.01 | 0.01 | 0.01 |
|  | Dose 1 | 0.03 | 0.03 | 0.03 | 0.03 |
|  | Dose 2 | 0.1 | 0.1 | 0.1 | 0.1 |
|  | Dose 3 | 0.3 | 0.3 | 0.3 | 0.3 |
|  | Dose 4 | 1.0 | 1.0 | 1.0 | 1.0 |
|  | Open dose | 2.5 | 4.0 | 5.0 | 5.5 |
|  | **Cumulative protein dose**** | **3.93** | **5.43** | **6.43** | **6.93** |
|  | Total Milk (g) | 116g | 160g | 189g | 204g |

**Table E2.** Severity criteria used: Practall (A), Ewan & Clark (B), and adrenaline use (C).

1. Practall Guidelines^1^

| **Symptoms and signs developed during oral food challenges (OFC)** | | **Criteria** | |
| --- | --- | --- | --- |
|  |  | Severe | Non-severe |
| SKIN | Erythematous rash | >3 | <=3 |
|  | Pruritus | 3 | 0,1,2 |
|  | Urticaria/angioedema | 3 | 0,1,2 |
|  | Rash | 3 | 0,1,2 |
| UPPER RESPIRATORY | Sneezing / itching | 3 | 0,1,2 |
| LOWER RESPIRATORY | Wheezing | 1,2,3 | 0 |
|  | Laryngeal | 2,3 | 0,1 |
| GASTROINTESTINAL | Subjective complaints | 3 | 0,1,2 |
|  | Objective complaints | 2,3 | 0,1 |
| CARDIOVASC  NEUROLOGIC |  | 1,2,3 | 0 |

1. Ewan & Clark^2^

| **Symptom score** | | **Severity classification** |
| --- | --- | --- |
| **1** | Localized cutaneous erythema  Localized urticaria  Localized angioedema  Oral pruritus | **Mild** |
| **2** | Generalized erythema  Generalized urticaria  Generalized angioedema |  |
| **3** | Gastrointestinal symptoms  Rhinitis | **Moderate** |
| **4** | Laryngeal edema  Mild asthma | **Severe** |
| **5** | Dyspnea  Hypotension |  |

1. Adrenaline use

| **Adrenaline used for treatment** | **Severity classification** |
| --- | --- |
| YES | Severe |
| NO | Non-Severe |

**Table E3.** Severity of symptoms during positive oral food challenges to baked milk according to the Practall guidelines, Ewan & Clark and use of adrenaline for treatment. Severity criteria are marked in red, according to the Practall guidelines^1^.

| **ID** | **SKIN** | | | | **Respiratory** | | | | **Gastro intestinal** | | | **Other** | **Ewan**  **& Clark** | **Treatm.** |
| --- | --- | --- | --- | --- | --- | --- | --- | --- | --- | --- | --- | --- | --- | --- |
|  | **% eryth.** | **Pruritus** | **Urticaria Angioed.** | **Rash** | **Rhinitis** | **Wheez.** | **Laryng. Sympt.** | **Subj. GI** | | **Obj. GI** | **Cardiol**  **Neurol** | |  | **AAI** |
|  | **>3** | **3** | **3** | **3** | **3** | **1,2,3** | **2,3** | **3** | | **2,3** | **1,2,3** | | **4,5** | **1** |
| 8 | 2 |  | 1 |  |  |  |  |  | |  |  | | 2 |  |
| 26 |  | 0 | 1 | 2 | 0 | 0 | 0 | 0 | | 0 | 0 | | 1 |  |
| 29 | **4** | **2** | **1** |  | **1** |  | **2** | **1** | |  |  | | **4** |  |
| 42 | **6** | **1** | **1** | **1** |  | **1** | **2** |  | |  |  | | **4** | **1** |
| 50 |  | **2** | **2** |  | **0** |  | **1** |  | |  |  | | **4** | **1** |
| 51 | **40** |  |  | **2** | **2** |  | **2** |  | |  |  | | **4** | **1** |
| 67 | **10** | **2** | **2** | **2** | **1** | **0** | **1** | **0** | | **0** | **0** | | **4** | **1** |
| 71 | 3 | 1 | 2 |  |  |  |  |  | |  |  | | 1 |  |
| 79 | 2 |  | 1 |  |  |  |  |  | |  |  | | 1 |  |
| 90 |  |  |  |  | 1 |  | 1 |  | |  |  | | 3 |  |
| 99 |  |  | 2 |  |  |  |  |  | |  |  | | 1 |  |
| 151 |  | 2 | 2 |  | 2 |  | 2 |  | |  |  | | 3 |  |
| 155 |  |  | 2 |  |  |  |  |  | |  |  | | 2 |  |
| 171 |  |  | 1 |  | 2 |  |  |  | |  |  | | 3 |  |
| 185 |  |  |  |  |  |  | **2** |  | |  |  | | **4** | **1** |
| 195 |  | 2 | 1 |  |  |  |  |  | |  |  | | 2 |  |
| 200 |  | 2 | 2 |  |  |  |  |  | |  |  | | 1 |  |
| 215 |  |  | 2 |  |  |  |  |  | |  |  | | 2 |  |
| 273 |  |  |  |  |  |  |  | 1 | |  |  | | 1 |  |
| 286 |  |  | **1** |  | **2** |  |  |  | |  |  | | **4** |  |
| 319 |  | 1 | 1 |  | 1 |  | 1 |  | |  |  | | 1 |  |
| 368 |  |  |  |  |  |  | 2 |  | |  |  | | 1 |  |

**Table E4.** Symptoms and signs developed during oral food challenges to baked (n=122) and fresh milk (n=49) performed as part of the BAT2 study and their severity, according to the Practall guidelines.

| **Symptoms and signs developed during oral food challenges (OFC)** | | | **Positive baked milk OFC (n=22)** | | **Positive fresh milk OFC (n=49)** | |
| --- | --- | --- | --- | --- | --- | --- |
| SKIN | Erythematous rash | 7 (32%) | | 8 (16%) | |  |
|  | Pruritus | 10 (46%) | | 17 (35%) | |  |
|  | Urticaria/angioedema | 17 (77%) | | 30 (61%) | |  |
|  | Rash | 4 (18%) | | 6 (12%) | |  |
| UPPER RESPIRATORY | Sneezing / itching | 10 (46%) | | 17 (35%) | |  |
| LOWER RESPIRATORY | Wheezing | 3 (14%) | | 2 (4%) | |  |
|  | Laryngeal | 11 (50%) | | 5 (10%) | |  |
| GASTROINTESTINAL | Subjective complaints | 4 (18%) | | 11 (22%) | |  |
|  | Objective complaints | 2 (9%) | | 5 (10%) | |  |
| CARDIOVASC  NEUROLOGIC |  | 2 (9%) | | 1 (2%) | |  |

* Abbreviations: OFC: Oral food challenges.

**Table E5. Basophil activation using different stimulants in children with allergic reactions to baked or fresh cow’s milk.**

**E.5.1. Severe versus non-severe reactors during OFC to baked or fresh milk, as defined by the Practall classification. P values refer to Mann-Whitney U test. *Abbreviations: BAT, Basophil activation test; anti-IgE, anti-immunoglobulin E.**

**A. BAKED MILK**

| **BAT (%CD63+ Basophils)** | **Severe reactors**  **(n=7)** | **Non-severe reactors**  **(n=15)** | **P value** |
| --- | --- | --- | --- |
| **No stimulant** | 2.37 (2.27; 3.39) | 1.95 (1.50; 2,35) | **0.020** |
| **0.1ng/ml milk extract** | 7.72 (4.02; 39.14) | 4.52 (0.55; 10.16) | 0.110 |
| **1ng/ml milk extract** | 13.52 (8.61; 31.47) | 8.56 (3.23; 17.69) | 0.110 |
| **10ng/ml milk extract** | 21.41 (17.73; 45.74) | 16.14 (6.26; 20.56) | **0.038** |
| **100ng/ml milk extract** | 37.0 (22.7; 59.9) | 19.5 (12.0; 29.6) | **0.020** |
| **1,000ng/ml milk extract** | 43.13 (23.81; 69.88) | 30.36 (13.61; 35.14) | **0.037** |
| **10,000ng/ml milk extract** | 57.63 (40.61; 75.38) | 30.62 (13.61; 42.02) | **0.007** |
| **0.1ng/ml baked milk** | 2.2 (0; 45.18) | 1.82 (0; 4.75) | 0.535 |
| **1ng/ml baked milk** | 5.23 (1.96; 58.48) | 2.98 (0.75; 9.72) | 0.287 |
| **10ng/ml baked milk** | 11.42 (4.86; 61.28) | 9.11 (1.36; 21.90) | 0.287 |
| **100ng/ml baked milk** | 24.12 (13.93; 70.58) | 16.23 (6.29; 26.27) | 0.110 |
| **1,000ng/ml baked milk** | 31.31 (20.03; 70.58) | 27.33 (18.79; 35.23) | 0.241 |
| **10,000ng/ml baked milk** | 45.72 (29.91; 80.07) | 29.58 (12.19; 45.03) | 0.067 |
| **Anti-IgE** | 53.22 (27.51; 69.97) | 42.95 (34.93; 69.91) | 0.877 |
| **fMLP** | 29.54 (21.01; 40.23) | 29.58 (15.38; 39.88) | 0.535 |
| **BAT (SI CD203c)** | **Severe reactors**  **(n=7)** | **Non-severe reactors**  **(n=15)** | **P value** |
| **0.1ng/ml milk extract** | 2.27 (1.80; 4.0) | 1.63 (1.07; 2.17) | 0.079 |
| **1ng/ml milk extract** | 3.06 (1.89; 3.10) | 1.99 (1.11; 2.92) | 0.149 |
| **10ng/ml milk extract** | 3.45 (2.82; 4.21) | 2.31 (1.21; 3.40) | 0.056 |
| **100ng/ml milk extract** | 4.51 (3.12; 5.61) | 3.20 (2.21; 4.27) | 0.056 |
| **1,000ng/ml milk extract** | 5.46 (3.18; 6.24) | 3.74 (1.82; 4.35) | 0.149 |
| **10,000ng/ml milk extract** | 5.77 (3.55; 6.74) | 4.13 (2.09; 5.09) | 0.094 |
| **0.1ng/ml baked milk** | 1.49 (1.08; 3.54) | 1.30 (1.00; 1.63) | 0.400 |
| **1ng/ml baked milk** | 2.69 (1.26; 3.41) | 1.32 (1.06; 1.87) | 0.128 |
| **10ng/ml baked milk** | 2.97 (1.62; 6.70) | 2.16 (1.26; 3.41) | 0.224 |
| **100ng/ml baked milk** | 4.52 (3.12; 7.10) | 2.98 (1.46; 3.92) | 0.128 |
| **1,000ng/ml baked milk** | 5.23 (2.72; 6.70) | 3.79 (1.73; 5.13) | 0.255 |
| **10,000ng/ml baked milk** | 5.13 (3.56; 6.63) | 3.89 (1.88; 5.42) | 0.255 |
| **Anti-IgE** | 4.95 (3.34; 6.52) | 4.86 (2.83; 5.77) | 0.636 |
| **fMLP** | 2.19 (1.71; 3.28) | 1.84 (1.38; 2.96) | 0.443 |

**B. FRESH MILK**

| **BAT (%CD63+ Basophils)** | **Severe reactors**  **(n=12)** | **Non-severe reactors (n=37)** | **P value** |
| --- | --- | --- | --- |
| **No stimulant** | 1.75 (1.13; 2.44) | 1.89 (1.29; 2.39) | 0.731 |
| **0.1ng/ml milk extract** | 2.19 (0; 4.44) | 1.17 (0.38; 3.06) | 0.893 |
| **1ng/ml milk extract** | 1.20 (0; 6.65) | 1.71 (0.45; 5.49) | 0.778 |
| **10ng/ml milk extract** | 3.12 (0.31; 14.71) | 3.31 (0.75; 7.98) | 0.932 |
| **100ng/ml milk extract** | 4.45 (1.0; 20.18) | 4.89 (1.38; 13.04) | 0.951 |
| **1,000ng/ml milk extract** | 10.19 (2.46; 37.51) | 14.75 (3.39; 36.37) | 0.598 |
| **10,000ng/ml milk extract** | 10.19 (2.46; 37.51) | 14.75 (3.39; 36.37) | 0.686 |
| **0.1ng/ml baked milk** | 0.58 (0; 2.28) | 0.03 (0; 1.96) | 0.686 |
| **1ng/ml baked milk** | 1.20 (0; 2.12) | 0.20 (0; 2.61) | 0.825 |
| **10ng/ml baked milk** | 1.04 (0.07; 3.01) | 0.70 (0; 4.80) | 0.864 |
| **100ng/ml baked milk** | 1.56 (0; 4.69) | 1.69 (0; 13.41) | 0.326 |
| **1,000ng/ml baked milk** | 1.50 (0; 12.38) | 4.17 (0.55; 21.08) | 0.320 |
| **10,000ng/ml baked milk** | 3.54 (2.60; 21.88) | 11.09 (5.51; 32.36) | 0.119 |
| **Anti-IgE** | 54.0 (12.46; 65.13) | 28.18 (13.17; 53.54) | 0.275 |
| **fMLP** | 23.36 (15.31; 62.40) | 44.60 (30.51; 64.75) | 0.131 |
| **BAT (SI CD203c)** | **Severe reactors**  **(n=12)** | **Non-severe reactors (n=37)** | **P value** |
| **0.1ng/ml milk extract** | 1.15 (1.0; 1.44) | 1.10 (1.01; 1.49) | 0.961 |
| **1ng/ml milk extract** | 1.20 (1.08; 1.30) | 1.24 (1.04; 1.65) | 0.932 |
| **10ng/ml milk extract** | 1.28 (1.12; 1.58) | 1.47 (1.10; 2.35) | 0.893 |
| **100ng/ml milk extract** | 1.53 (1.12; 2.37) | 1.70 (1.12; 2.60) | 0.825 |
| **1,000ng/ml milk extract** | 1.76 (1.25; 3.54) | 1.77 (1.35; 2.68) | 0.990 |
| **10,000ng/ml milk extract** | 1.76 (1.17; 3.43) | 2.53 (1.41; 3.74) | 0.371 |
| **0.1ng/ml baked milk** | 1.05 (0.98; 1.14) | 1.02 (0.98; 1.14) | 0.922 |
| **1ng/ml baked milk** | 1.02 (0.98; 1.28) | 1.08 (0.99; 1.27) | 0.404 |
| **10ng/ml baked milk** | 1.05 (0.97; 1.28) | 1.08 (1.0; 1.42) | 0.686 |
| **100ng/ml baked milk** | 1.12 (0.99; 1.53) | 1.35 (1.10; 0.81) | 0.091 |
| **1,000ng/ml baked milk** | 1.28 (1.04; 1.63) | 1.85 (1.21; 3.09) | 0.095 |
| **10,000ng/ml baked milk** | 1.50 (1.23; 2.06) | 2.37 (1.51; 3.92) | 0.079 |
| **Anti-IgE** | 4.20 (1.96; 5.61) | 3.36 (1.93; 5.80) | 0.704 |
| **fMLP** | 2.22 (1.51; 3.64) | 2.80 (1.03; 4.63) | 0.358 |

**Table E.5.2. Lower versus higher threshold of reactivity to baked or fresh milk during OFC. P values refer to Mann-Whitney U test. *Abbreviations: BAT, Basophil activation test; anti-IgE, anti-immunoglobulin_E.**

**A. BAKED MILK**

| **BAT (%CD63+ Basophils)** | **Threshold </=0.44g (n=14)** | **Threshold >0.44g (n=8)** | **P value** |
| --- | --- | --- | --- |
| **No stimulant** | 2.27 (1.62; 2.56) | 2.56 (1.81; 3.16) | 0.547 |
| **0.1ng/ml milk extract** | 7.58 (3.01; 17.39) | 3.52 (0.43; 9.96) | 0.336 |
| **1ng/ml milk extract** | 11.93 (6.13; 20.42) | 10.29 (2.36; 16.76) | 0.750 |
| **10ng/ml milk extract** | 17.73 (12.03; 31.50) | 18.79 (21.65; 22.28) | 0.860 |
| **100ng/ml milk extract** | 20.93 (16.70; 36.28) | 27.0 (14.12; 37.39) | 0.804 |
| **1,000ng/ml milk extract** | 30.87 (18.67; 41.85) | 33.68 (16.27; 40.31) | 1.0 |
| **10,000ng/ml milk extract** | 32.05 (14.85; 54.53) | 39.82 (23.63; 48.67) | 0.860 |
| **0.1ng/ml baked milk** | 2.22 (0.26; 5.20) | 1.02 (0; 8.88) | 0.50 |
| **1ng/ml baked milk** | 5.23 (1.02; 11.66) | 2.99 (0; 13.90) | 0.374 |
| **10ng/ml baked milk** | 11.42 (3.52; 20.90) | 9.11 (1.30; 26.59) | 0.804 |
| **100ng/ml baked milk** | 15.88 (9.43; 27.27) | 20.41 (7.29; 41.16) | 0.645 |
| **1,000ng/ml baked milk** | 27.98 (18.58; 35.31) | 30.42 (21.77; 41.32) | 0.521 |
| **10,000ng/ml baked milk** | 33.22 (14.94; 54.02) | 38.49 (26.62; 46.31) | 0.678 |
| **Anti-IgE** | 46.60 (33.05; 67.16) | 56.08 (30.82; 70.34) | 0.734 |
| **fMLP** | 27.72 (17.09; 40.03) | 31.87 (13.29; 46.21) | 0.804 |
| **BAT (SI CD203c)** | **Threshold </=0.44g (n=14)** | **Threshold >0.44g (n=8)** | **P value** |
| **0.1ng/ml milk extract** | 1.86 (1.27; 2.68) | 1.92 (1.12; 2.24) | 0.916 |
| **1ng/ml milk extract** | 2.55 (1.32; 3.10) | 2.37 (1.32; 2.99) | 0.972 |
| **10ng/ml milk extract** | 2.69 (1.99; 3.94) | 2.97 (1.12; 3.89) | 0.916 |
| **100ng/ml milk extract** | 3.43 (2.23; 5.12) | 4.31 (2.70; 4.97) | 0.374 |
| **1,000ng/ml milk extract** | 3.57 (2.23; 5.12) | 4.10 (3.02; 5.96) | 0.374 |
| **10,000ng/ml milk extract** | 4.12 (2.73; 5.68) | 4.71 (3.70; 6.89) | 0.301 |
| **0.1ng/ml baked milk** | 1.34 (1.04; 1.71) | 1.34 (1.03; 1.90) | 0.916 |
| **1ng/ml baked milk** | 1.57 (1.21; 2.71) | 1.26 (1.05; 2.50) | 0.456 |
| **10ng/ml baked milk** | 2.81 (1.39; 3.23) | 2.42 (1.34; 4.37) | 0.860 |
| **100ng/ml baked milk** | 3.12 (1.70; 4.17) | 4.01 (2.45; 6.40) | 0.301 |
| **1,000ng/ml baked milk** | 3.25 (2.26; 4.81) | 5.21 (3.42; 6.85) | 0.121 |
| **10,000ng/ml baked milk** | 3.71 (2.28; 4.72) | 5.54 (3.69; 7.42) | 0.076 |
| **Anti-IgE** | 4.03 (3.19; 5.37) | 5.59 (3.68; 8.27) | 0.210 |
| **fMLP** | 1.91 (1.48; 2.73) | 2.20 (1.50; 3.94) | 0.645 |

**B. FRESH MILK**

| **BAT (%CD63+ Basophils)** | **Threshold <=0.143g (n=25)** | **Threshold >0.143g (n=24)** | **P value** |
| --- | --- | --- | --- |
| **No stimulant** | 1.79 (1.17; 2.15) | 2.04 (1.40; 2.47) | 0.190 |
| **0.1ng/ml milk extract** | 1.29 (0.54; 5.74) | 0.96 (0; 2.70) | 0.151 |
| **1ng/ml milk extract** | 2.18 (0.75; 7.19) | 0.50 (0; 4.38) | **0.036** |
| **10ng/ml milk extract** | 4.19 (1.13; 11.73) | 2.86 (0.31; 6.47) | 0.149 |
| **100ng/ml milk extract** | 6.53 (2.83; 24.28) | 3.23 (0.91; 9.09) | **0.017** |
| **1,000ng/ml milk extract** | 6.98 (5.10; 31.97) | 5.70 (2.35; 19.89) | 0.317 |
| **10,000ng/ml milk extract** | 17.31 (5.21; 37.27) | 10.19 (2.46; 35.70) | 0.358 |
| **0.1ng/ml baked milk** | 0 (0; 1.68) | 0.42 (0; 2.28) | 0.176 |
| **1ng/ml baked milk** | 0 (0; 1.29) | 1.26 (0; 3.29) | 0.176 |
| **10ng/ml baked milk** | 0.17 (0; 3.82) | 1.04 (0; 3.81) | 0.386 |
| **100ng/ml baked milk** | 1.20 (0; 19.14) | 2.22 (0.27; 7.94) | 0.942 |
| **1,000ng/ml baked milk** | 4.17 (1.47; 25.99) | 1.50 (0.25; 17.18) | 0.201 |
| **10,000ng/ml baked milk** | 12.11 (6.86; 40.94) | 5.57 (2.80; 24.90) | 0.078 |
| **Anti-IgE** | 41.15 (11.55; 52.85) | 25.27 (13.54; 61.28) | 0.828 |
| **fMLP** | 38.60 (20.86; 63.17) | 44.43 (28.89; 63.81) | 0.599 |
| **BAT (SI CD203c)** | **Threshold <=0.143g (n=25)** | **Threshold >0.143g (n=24)** | **P value** |
| **0.1ng/ml milk extract** | 1.22 (1.02; 1.65) | 1.03 (1.0; 1.27) | **0.029** |
| **1ng/ml milk extract** | 1.49 (1.13; 1.93) | 1.14 (1.03; 1.28) | **0.013** |
| **10ng/ml milk extract** | 1.58 (1.23; 2.40) | 1.23 (1.09; 1.50) | 0.056 |
| **100ng/ml milk extract** | 2.01 (1.44; 3.54) | 1.33 (1.10; 1.88) | **0.023** |
| **1,000ng/ml milk extract** | 2.08 (1.47; 3.50) | 1.49 (1.21; 2.49) | 0.107 |
| **10,000ng/ml milk extract** | 2.52 (1.59; 4.27) | 2.42 (1.24; 3.28) | 0.403 |
| **0.1ng/ml baked milk** | 1.03 (0.97; 1.11) | 1.02 (0.98; 1.14) | 0.508 |
| **1ng/ml baked milk** | 1.02 (0.98; 1.12) | 1.08 (1.01; 1.32) | 0.157 |
| **10ng/ml baked milk** | 1.06 (0.98; 1.20) | 1.15 (1.0; 1.30) | 0,522 |
| **100ng/ml baked milk** | 1.29 (1.07; 1.81) | 1.38 (1.06; 1.62) | 0,853 |
| **1,000ng/ml baked milk** | 1.91 (1.21; 3.59) | 1.33 (1.07; 2.47) | 0.117 |
| **10,000ng/ml baked milk** | 2.56 (1.52; 3.92) | 1.67 (1.39; 3.16) | 0.176 |
| **Anti-IgE** | 3.45 (1.95; 5.99) | 3.87 (1.81; 5.58) | 0.926 |
| **fMLP** | 2.37 (1.95; 4.38) | 2.80 (2.07; 4.48) | 0.695 |

**Table E6.** Diagnostic performance of optimal, 100% sensitivity and 100% specificity cut-offs for the second-best biomarkers for severity and threshold of allergic reactions to baked milk and fresh milk during OFC.

**SEVERITY**

| **Food and Parameter** | **Cut-off** | | **AUC ROC** | **Sensitivity** | **Specificity** | **PPV** | **NPV** | **Accuracy** | **TP/FP** | **TN/FN** |
| --- | --- | --- | --- | --- | --- | --- | --- | --- | --- | --- |
| BAKED MILK  %CD63+ Basophils at 100ng/ml of milk extract | 100% S | 13.97 | 0.643 | 100% | 29% | 41% | 100% | 52% | 7 / 10 | 4 / 0 |
|  | OPTIMAL | 35.14 | 0.822 | 71% | 93% | 83% | 87% | 86% | 5 / 1 | 13 / 2 |
|  | 100% Sp | 49.19 | 0.715 | 43% | 100% | 100% | 78% | 81% | 3 / 0 | 14 / 4 |

**THRESHOLD**

| **Food and Parameter** | **Cut-off** | | **AUC ROC** | **Sensitivity** | **Specificity** | **PPV** | **NPV** | | **Diagnostic accuracy** | **TP/FP** | **TN/FN** | |
| --- | --- | --- | --- | --- | --- | --- | --- | --- | --- | --- | --- | --- |
| **FRESH MILK**  SI CD203c ME 1ng/ml | 100% S | 0 | 0.5 | 100% | 0% | 53% | - | | 53% | 25 / 22 | 0 / 0 | |
|  | OPTIMAL | 1.30 | 0.732 | 60% | 86% | 83% | 66% | | 72% | 15 / 3 | 19 / 10 | |
|  | 100% Sp | 2.48 | 0.5 | 16% | 100% | 100% | | 51% | 55% | 4 / 0 | 22 / 21 |  |

*****Abbreviations: AUC: Area under the curve; BAT: Basophil activation test; FN: False negative; FP: False positive; IgE: Immunoglobulin E; NPV: Negative predictive value; PPV: Positive predictive value; ROC: Receiver operating characteristics curve; S: Sensitivity; Sp: Specificity; TN: true negative; TP: true positive.

**Figure E1.** Severity of symptoms experienced during challenges to baked milk (A, n=22) and fresh milk (B, n=49), assessed and classified in real-time by the clinical team attending the oral food challenge,. Scores from 0 to 3 are given depending on the severity of symptoms, according to the Practall guidelines^16^.

**Figure E2.** Receiver Operating Characteristic curve for the basophil activation test to predict severe reactions during oral food challenges to baked milk using %CD63+ Basophils at 100ng/ml of milk extract.

**E-References**

1. Sampson HA, Gerth van Wijk R, Bindslev-Jensen C, et al. Standardizing double-blind, placebo-controlled oral food challenges: American Academy of Allergy, Asthma & Immunology-European Academy of Allergy and Clinical Immunology PRACTALL consensus report. J Allergy Clin Immunol 2012;130(6):1260-74. DOI: 10.1016/j.jaci.2012.10.017.

2. Ewan PW, Clark AT. Long-term prospective observational study of patients with peanut and nut allergy after participation in a management plan. Lancet 2001;357(9250):111-5. DOI: 10.1016/s0140-6736(00)03543-1.
